# A Robust and Data-Efficient Deep Learning Model for Cardiac Assessment without Segmentation

**DOI:** 10.21203/rs.3.rs-5290766/v1

**Published:** 2024-10-28

**Authors:** Conor M. Artman, Ricardo Henao

**Affiliations:** 1 AI Research Group, Lawrence Livermore National Laboratory, 7000, East Avenue, Livermore, 94550, California, United States.; 2 Department of Biostatistics & Bioinformatics, Duke University, 2424, Erwin Road, Suite 1104, Durham, 27705, North Carolina, United States.

**Keywords:** Ultrasounds, Computer Vision, Clinical Decision Support, Robust Deep Learning, Cardiac Assessment

## Abstract

Video-based deep learning (DL) algorithms often rely on segmentation models to detect clinically important features in transthoracic echocardiograms (TTEs). While effective, these algorithms can be too data hungry for practice and may be sensitive to common data quality issues. To overcome these concerns, we present a data-efficient DL algorithm, Scaled Gumbel Softmax (SGS) EchoNet, that is robust to these common data quality issues and, importantly, requires no ventricular segmentation model. In lieu of a segmentation model, we decompose and transform the output of an R(2+1)D convolutional encoder to estimate frame-level weights associated with the cardiac cycle, that are then used to obtain a video representation that can be used for estimation. We find that our transformation obviates the need for a segmentation model while improving the ability of the predictive model to handle noisy inputs. We show that our model achieves comparable performance to the state of the art, while demonstrating robustness to noise on an independent (external) validation set.

## 1 Introduction

Diagnostic imaging in healthcare is essential for disease identification, treatment, disease management, and prognosis. While there are various imaging modalities available to clinicians, ultrasound imaging is widespread and convenient because it is cost-effective and can provide portable real-time imaging, *e.g*., it can be easily deployed to urban and rural hospital bedsides, developing countries, or emergency scenarios [1]. In contrast to modalities such as radiography or computerized tomography, TTEs do not use ionizing radiation, which can be harmful to patients after repetitive exposure [2]. Consequently, ultrasonography has grown into many medical specialties ranging from neurosurgery to cardiology, dentistry, and oncology [3].

Despite widespread use, ultrasound imaging quality is affected by machine-level, operator-level, and patient-level variation. Specifically, ultrasound machines tend to produce images with variable quality within and across manufacturers [4, 5]. Further, ultrasound imaging quality heavily depends on the experience level of the operator, thus there is high inter-operator variability [6]. Finally, ultrasound image quality can suffer from random noise and imaging artifacts due to issues ranging from electromagnetic interference to heavy sweating on a patient’s skin [7, 8]. Examples of such variation include dark spots, reverberations distorting the image, blurry images, random white specks interfering with images, or brightness issues, *i.e*., images that are too dark or bright [8]. Altogether, these three sources of variation can seriously impair clinicians’ ability to make objective and accurate decisions. Therefore, decision support tools for automatic ultrasound analysis must be designed with these sources of variation in mind.

Image reliability is especially important in diagnosing cardiomyopathy because clinicians may need to quantify several metrics from an ultrasound. In turn, each metric determines the necessary cross-section view of a heart, generically referred to as a view. For example, it is recommended that clinicians use the parasternal long-axis (PLAX) view for linear measurements of the left-ventricle’s walls [9]. For volumetric measurements, left ventricular ejection fraction (EF) helps clinicians decide if patients are eligible for life-prolonging therapies. Further, they use apical two-chamber (AP2) and apical four-chamber (AP4) views for EF measurements because they allow clinicians to see the entire left ventricle [9]. They recommend using both the apical 4-chamber (AP4) and the apical 2-chamber (AP2) views as a guideline, but EF is also measurable from a single AP4 view ultrasound. In this paper, we work solely with AP4 views for comparability between our model and a previously proposed model with associated publicly available data [10].

Unfortunately, determining EF from AP4 views is labor-intensive and subject to inter-rater variability. Reports show that despite advances in medical machinery and significant training for clinicians reading EF, inter-rater variation can range from 7.6% to 13.9% [11]. While this amount of variation may appear small, other reports have shown that patients with a minor drop in EF have medically significant increases in morbidity and mortality [12]. Together with the high volume of cases in the United States, these reports underscore that small differences in EF values caused by imprecise segmentation may cause serious negative outcomes for patients at scale [13]. Therefore, there is an opportunity for sophisticated decision support tools to supplant tedious tasks for clinicians and provide fast, robust, and data-efficient estimates for clinically significant quantities in cardiology [14].

For EF estimation, the best-performing approaches incorporate state-of-the-art segmentation models into a two-model approach: one for end systole (ES) and end diastole (ED) phase identification and a separate model for EF estimation. After fitting both models, researchers then devise a way to use the segmentation results to select individual cardiac beats to be used as inputs to the EF estimation model [10, 15–19] (see Section 2 for details about related work). Such approach usually involves the estimation of left ventricle area (or volume) as a means to identify frames consistent with ES and ED.

It is our view that composite, two-model approaches for estimating EF may inadvertently result in hospital-to-hospital performance vulnerabilities. First, regardless of the segmentation model, segmentation labels may be poor in quality or unavailable due to cost and time constraints, so (semi-) supervised transfer or refinement of a segmentation model may be difficult or impossible. When view segmentation labels are available but potentially mislabeled, refining a segmentation model on TTEs from a new health care system could cause worse segmentation performance, *e.g*., through phenomena such as catastrophic forgetting [20]. Second, and as a direct consequence, a sensitive segmentation model will cause miscalibration in the EF estimation model, resulting in poor EF estimation performance.

Motivated by these concerns, in this paper we consider the problem of estimating EF from AP4 view TTEs without a segmentation model and when view labels may be mislabeled. We propose a parsimonious and robust DL algorithm termed Scaled Gumbel Softmax (SGS) EchoNet that only requires a single model for video-based EF estimation. Our model consists of three components. First, we use an R (2+1) D CNN encoder [21] that takes in AP4 view TTEs. We use an R(2+1)D CNN because interleaved 2D and 1D convolutional blocks capture spatiotemporal relationships in videos with fewer parameters than 3D convolutions, lower computational cost, and without loss of performance [21].

Second, our model aggregates TTE representations by decomposing and transforming the output of the R(2+1)D encoder. Specifically, the model automatically up-weights frame-level features near ED and ES phase frames before estimating EF. Note that this module stands in for the role of a segmentation model.

Third, our model passes the aggregated features to a decoder that reshapes these features and forwards them to a final linear layer for EF estimation.

To the best of our knowledge, [22] proposes the only other approach that identifies ED/ES frames without a segmentation model for the purpose of estimating EF. While their methodology constitutes an important proof-of-concept, their quantitative results for EF estimation are not competitive with models that use segmentation models, *e.g*., EchoNet and EchoGraph, whereas our approach achieves results that are comparable to the state-of-the-art. In [23], they also develop a method for ED/ES frame detection, but they do not consider EF estimation as downstream task. We note that one commonality between [22], [10], [19], and our work is that all four rely on a R (2+1)D network for encoding spatiotemporal features from an AP4 ultrasound. Importantly, none of the other works, however, study their models outside the data released with [10]. Consequently, EchoNet, EchoGraph, and [22] models rely on the availability well-labeled ultrasound frames for their segmentation models. In this paper, we explicitly study the performance of the proposed model in the data released by [10] and on an external validation set under varying levels of view label noise and common (realistic) data quality issues.

The rest of this paper is laid out as follows. In Section 2, we discuss related work. In Section 3, we introduce ejection fraction estimation, data collection details, and image pre-processing. In Section 4, we formalize the proposed model and the models we compare it to. Finally, we present quantitative results in Section 5 and conclude the paper in Section 6.

## 2 Related Work

The primary difficulty for EF estimation in human- or computer-based methods is correctly identifying individual beats by analyzing the ED and ES phases of the cardiac cycle via TTE frames. Specifically, the length of the TTE, in number of frames, is directly associated with the number of beats, *i.e*., how many cardiac cycles are represented, each of which will have corresponding ED and ES frames. In general, the identification of these frames is more difficult in shorter sequences with fewer or incomplete cardiac cycles. Consequently, the TTE may not be able to accurately capture the maximum and minimum left-ventricular volumes, thus the frames associated with ED and ES may be incorrectly assigned.

Next, the choice of EF estimation methodology influences how the quantity of data affects ED and ES phase identification. For example, though convolutional neural networks (CNNs) work well for segmentation tasks, there may not be enough ground-truth labels, also called *tracings*, for successful (semi-)supervised learning ^1^. For ventricular segmentations, this is because CNNs need tracings to learn how to segment the correct area of a heart ultrasound. However, as mentioned, tracings can be difficult or costly to acquire because clinicians must draw frame-by-frame outlines of a specific area of the heart (see Section 3.4 for discussion).

While CNN encoders constitute the state-of-the-art for segmentation tasks, they are not the only option for segmenting TTEs or predicting important quantities for cardiac assessment. Before CNNs became popular, many segmentation models used shape priors to bound the segmentation area, *e.g*., active contours, deformable models, active shape models, or active appearance models [3]. More narrowly, we draw inspiration from work outside of the CNN literature that focuses on estimating ED and ES phases from subsets of a TTE’s B-mode images.

While various techniques have been explored for estimating ED and ES phases from B-mode images, *e.g*., with manifold learning, dimension reduction, or other methods [3,10,24−27], one important example that we rely on in this work is [23]. This paper recasts the ED and ES phase identification problem as a non-negative matrix factorization (NMF) problem. In precise terms, given a non-negative matrix X, NMF attempts to estimates two non-negative matrices, say W and H, that satisfy X≈WH by iteratively minimizing the error between X and WH. The resulting columns of W are the basis vectors capturing underlying features in the data, while the columns of H represent weights associated with W‘s basis vectors in the reconstruction of the original data. This can be implemented by applying various optimization techniques, e.g., alternating least squares. In [23], after reshaping each frame of an TTE into a column of pixels stacked into a matrix, they apply an NMF algorithm to estimate ED and ES cycles.

After U-Net-style architectures propelled progress in left-ventricular segmentation and eclipsed previous performances for 2D and 3D TTEs, CNNs have dominated segmentation tasks when ground-truth labels, such as tracings, are available [10, 26–28]. Most recently, nnU-Net has emerged as a state-of-the-art model for automated medical segmentation tasks. nnU-Net provides an out-of-the-box solution for semantic segmentation by integrating automatic pre-processing, network encoder selection, training, and post-processing functions [29]. In effect, nnU-Net leverages dataset characteristics for automatic, dataset-specific hyperparameter optimization. Impressively, nnU-Net improves on most existing methods on 23 datasets without any human intervention [29]. In [30], they propose TransUNet as a model for general medical segmentation that combines the strengths of Transformers with a U-Net style architecture. TransUNet tokenizes image patches from a convolutional feature map and uses them as input sequences. Its decoder up samples encoded features and combines them with higher-resolution CNN feature maps to precisely capture finer local contexts.

For two-model approaches, EchoNet Dynamic (EchoNet) [10] and EchoGraph [19] achieve state-of-the-art EF estimation performance by imitating clinicians’ workflow (see Section 4 for EchoNet details). Out of concern for computational expense and robustness, EchoGraph uses a graph convolutional network (GCN) to segment the left ventricle by detecting anatomical structure with key points, *i.e*., frame-by-frame pixel locations which bound the left ventricle’s area. These key-points estimated by the GCN and the features estimated by the EF model are used as features for a final EF prediction. The result is a two-model algorithm that is faster to train and produces fewer segmentation errors than EchoNet. Both models are constructed to be conceptually explainable to practitioners, but the trade-off is that they require fitting two separate DL models for segmentation and then EF estimation.

## 3 Materials and Methods

We work with a public dataset collected by [10] through Stanford Health Care and one from Duke Health Care. First we review EF estimation. Then we explain how Stanford and Duke Health Care collected their TTEs. From there, we elaborate on common data quality issues in practice. Finally, we close this section with data pre-processing details for both datasets.

### 3.1 Estimation of Ejection Fraction

When using EF to set a diagnostic threshold for cardiomyopathy, clinicians must hand-label TTEs to identify end-systolic (ES, contraction phase) and end-diastolic (ED, expansion phase) volumes. ES can be identified as the frame after the aortic valve closes *or* the time point where left-ventricular volume is smallest. ED can be identified as the frame after the mitral valve closes or as the time point *or* frame when the left ventricular (LV) volume is largest [9]. Clinicians then use these labels to calculate the difference between ED volume (EDV) and ES volume (ESV), also called stroke volume (SV), and divide by EDV to obtain EF. A lower EF indicates higher risk of cardiomyopathy, but in practice, a cutoff of 50% is common for flagging patients [12,31]. Therefore, identifying ES and ED phases is important to estimating EF, which is necessary for diagnosing cardiomyopathy. Formally, EF is written as follows

$$EF=100\left[\frac{EDV-ESV}{EDV}\right]=100\left[\frac{SV}{EDV}\right].$$

Note that inter-rater variability in ED and ES frames and volumes among different clinicians (and with disparate levels of expertise) can make both tracings and EF estimation imprecise. Compounded with the variability of an ultrasound machine’s imaging quality, EF estimates may be too variable for high-consequence medical decisions.

### 3.2 Stanford Health Care Ultrasound Data

An apical 4-chamber view shows each of the four chambers of the heart, with the ventricles on top and the atria on the bottom. Each apical 4-chamber (AP4 or A4C) TTE in our data corresponds to an unique patient study from 10, 030 individuals between 2016 and 2018 during standard clinical care at Stanford Health. Videos were generated with iE33, Sonos, Acuson SC2000, Epiq 5G or Epiq 7C ultrasound machines and the tabulated number of TTEs generated for each machine is not available. All 10,030 videos were randomized and split into 7,465 training videos, 1,277 validation videos, and 1,288 test videos by [10]. For our studies, we follow the same randomized allocation of videos to training, validation, and test sets to allow for direct comparison of performance. Data in [10] were de-identified for public use.

For the segmentation task, [10] provide manually labeled frames of the left ventricle during end-systole and end-diastole. As noted in [10], the average video contains 176 frames but only 2 labeled frames for weakly supervised segmentation for a total of 20, 060 weak frame-level segmentation labels. We refer the reader to [10] for additional details about the dataset.

### 3.3 Duke Health Care Ultrasound Data

We refer to the external validation set provided by Duke Health Care as the CATHeterization GENetics (CATHGEN) dataset. For further details about the study see [32]. 898 TTEs were collected with iE33 machines, 105 with Vivid 7, 26 with Logiq 7, and 15 were collected with Sonos machines for a total of 1, 044 TTEs.

There are no view labels available for the CATHGEN videos, so we train a view classifier to identify TTE views on a separate dataset and extract AP4 videos from the classified CATHGEN dataset afterward (see Section 3.5 for a description of our view classifier). We obtain a total of 1, 044 AP4 2D grayscale videos. Despite starting from a large dataset of 1,044AP4 videos, however, the number of usable views was relatively small for our validation set (see Table 1 for a breakdown of video numbers).

Comparing Stanford and CATHGEN video lengths, the average Stanford video is over four times longer than the average CATHGEN video. Stanford TTEs have 176 frames on average with a standard deviation of 60 and inter-quartile range of 55, while the average number of frames in CATHGEN TTEs was 41 frames with a standard deviation of 56 frames and an inter-quartile range of 18 frames. The shorter video length makes it a challenging external dataset because there are fewer cardiac cycles per video. Importantly, unlike the Stanford dataset, CATHGEN does not come with view or segmentation labels. Therefore, we ran EchoNet’s segmentation model as-is and used the resulting segmentations in EchoNet’s workflow for generating EF predictions on the CATHGEN TTEs in Section 5.5.

Since the CATHGEN dataset does not come with view labels, we use a separate dataset from the PROMISE study by [33], for which view labels are available. We then used these TTEs and their view labels to train our view classifier. In total, there were 8,899 DICOM files for which 5,700 TTEs had views. We randomly assigned these TTEs to train, validation, and test sets in approximately a 60/20/20% split. The training set had 3,455 videos, the validation set had 1, 137, and the test set had 1,108. Information for all 5,700 TTEs is as follows: 5,156 iE33 machines, 362 Vivid 7,149 Logiq 7, 33 Sonos machines. On average, each PROMISE TTE had roughly 46 frames per TTE with a standard deviation of approximately 47 frames and an inter-quartile range of 29 frames. See Table 2 for a breakdown of the views.

### 3.4 Data Quality Challenges

Despite data collection protocols, quality issues are common from one TTE dataset to another. One source of error can be differences in equipment quality. Human errors such as misuse of equipment, labeling errors, and clerical errors in recording EF are also likely to cause issues [34]. Additionally, flexibility in echocardiography guidelines can inadvertently contribute to data quality issues in practice. For example, if clinicians observe variation in a patient’s cardiac cycle, the American Society of Echocardiography (ASE) and the European Association of Cardiovascular Imaging (EASCVI) recommend tracing and then averaging up to 5 consecutive cardiac cycles [9]. Meanwhile, clinicians frequently evaluate EF from tracings of only a single beat; if a tracing is deemed inaccurate, clinicians can decide to visually approximate the EF [9]. These differences in tracing versus visual inspection are likely related to time or resource constraints on clinical staff, *e.g*., underfunded health systems may be understaffed, so clinicians may be rushed, may prefer visual inspections, or may be unavailable for data collection altogether.

In the CATHGEN dataset, we found that some patients’ EFs were imprecisely recorded. We observed that clinicians treated the EF value 55 as a binary variable, i.e., in cases where EF was ≥55, the value was recorded as 55. In addition to lacking segmentation and view labels, a significant number of TTEs in the Cathgen dataset contained a rendered Doppler feature overlaid on the TTE (see Table 1 for details). In grayscale, the Doppler overlay appears as a white cloud that flashes over important features in the TTE, and in full color, it appears as an evolving flash of red, yellow, white, and blue.

Finally, while DL approaches handle view classification tasks well, they may still misclassify views (we refer the reader to [35] for comparisons of various view classifiers and their performances). In this case, the segmentation or EF estimation model could receive a video which is outside its training set, because it may show the model another view of the heart that it has not been trained on. We explore this setting in Section 5.

### 3.5 Data Pre-Processing

In this section, we discuss how we pre-process our data to arrive at our final training, validation, and test sets for the Duke and Stanford Health Care Systems datasets.

#### Stanford Healthcare Systems Dataset

Each video was cropped and masked to remove text and all other information outside of the scanning sector, e.g., electrocardiogram information and respirometer information. The images were 600 × 600 or 768 × 768 pixels, depending on the ultrasound machine. The images were then down-sampled by cubic interpolation using OpenCV to produce standardized 112 × 112 pixel videos. All other quality control, de-identification, IRB procedures and demographic information are available in [10].

#### Duke Healthcare Systems Dataset (CATHGEN)

**Fig. 1:**
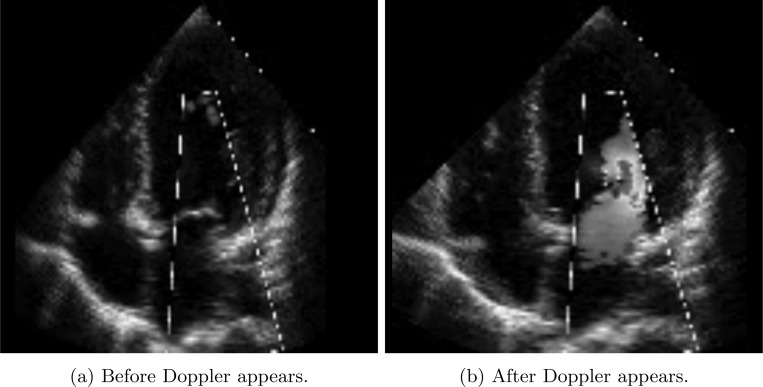
Example of TTE with Doppler.

For comparability, we apply the same pre-processing steps to Duke TTE videos with one extra step. After applying the Stanford pre-processing steps, we automatically screen out TTEs with Doppler present by filtering on attribute (0028,0014) when it is set to 1. (see Figure 1 for an example of when (0028,0014) is 1 ). Table 1 tallies the number of videos over by AP4 classification probability, the presence of videos with EF approximated to *EF=*55, and the presence of Doppler.

#### Duke View Classification Dataset (PROMISE)

We used the PROMISE Echo dataset to train our view classifier. Each video in the dataset is cropped and masked to remove text, electrocardiogram data, and any other information outside of the scanning zone. All videos are grayscale or converted to grayscale. From there, the pre-processing steps were the same as for the CATHGEN data.

We used the PROMISE Echo dataset to train a R(2+1)D-18 classification model, as described in [21]. Broadly, it is a R(2+1)D encoder pretrained on the Kinetics-400 dataset using a final softmax layer for classification and a cross-entropy loss function with a stochastic gradient descent optimizer [21,36]. After training and validating, we applied the classifier to the CATHGEN data to extract videos classified with an AP4 view.

## 4 Models

### 4.1 EchoNet Dynamic

**Fig. 2:**
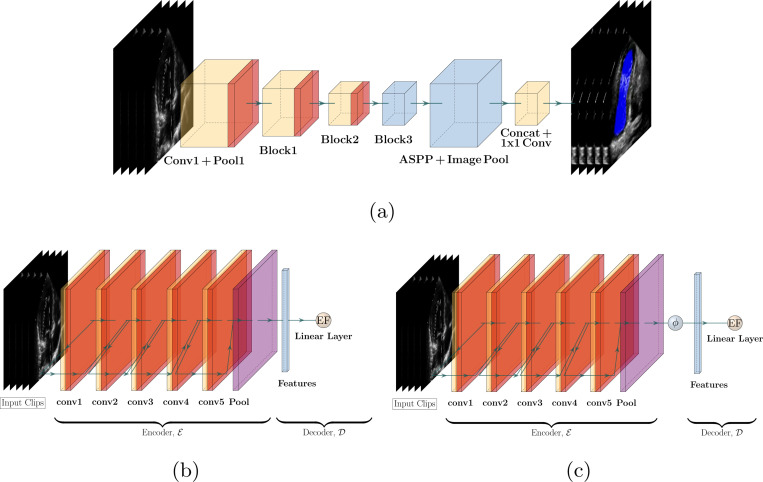
Model Diagrams. EchoNet Dynamic consists of two arms: (a) A DeepLabV3 encoder for LV segmentation and (b) an R(2+1)D encoder followed by spatiotemporal pooling for EF estimation. The segmentation model is used to identify and feed clips of the cardiac cycle to the EF estimation model. When we use EchoNet Dynamic without its segmentation model for clip selection, it is identical to (b) and we refer to it as EchoNet No-Seg. (c) Spectral EchoNet also uses a R(2+1)D encoder, but followed by spatial pooling and frame-level aggregation. All EF models use a linear layer after pooling.

EchoNet is an EF estimation and image segmentation model that is comprised of three components, with each component imitating a key step of a clinician’s cardiac assessment workflow [10]. The first component is a semantic segmentation model using the Deeplabv3 encoder [37]. Deeplabv3 uses a 50-layer ResNet base encoder with atrous convolutions to produce frame-level semantic segmentation of the left ventricle (LV). After initializing with random weights, [10] optimize their segmentation model with a pixel-level binary cross-entropy loss function using a stochastic gradient descent optimizer (see Figure 2a). The second EchoNet component is an R(2+1)D convolutional encoder that combines residual connections and interleaved spatial and temporal convolutions to obtain representation of the video input (see Figure 2b). This representation is aggregated via spatiotemporal pooling before being fed to a linear layer for EF estimation [21, 38]. The R(2+1)D model is initialized with pretrained weights from the Kinetics-400 dataset [36]. The final component uses the segmentation model’s output to identify each cardiac cycle in the ultrasound, generates a 32-frame clip for each cycle, estimates EF for each clip, and then averages all the EF estimates to produce EchoNet Dynamic’s final EF estimate.

### 4.2 EchoNet Dynamic without Segmentation

We refer to EchoNet Dynamic without its segmentation model as either EchoNet Dynamic No-Seg (EDNS) or simply EchoNet No-Seg. The latter uses the entire apical four-chamber view ultrasound video as input and estimates EF *without* the need for a segmentation model. Otherwise, it is identical to EchoNet and trained the same way. This approach is considered to mimic situations in which there is a need to refine the model with a new dataset (of TTEs and ground truth EF values) but LV segmentation labels are not available.

### 4.3 Spectral and Scaled Gumbel-Softmax EchoNet

Next we discuss how to approximate ED/ES phases without a segmentation model and describe our proposed model. We first develop Spectral EchoNet and then extend it to our final model, Scaled Gumbel Softmax (SGS) EchoNet.

When inspecting an AP4 TTE, it is visually clear that the primary source of variation is the cardiac cycle. One way to apply this observation is to build a segmentation model that captures the cardiac cycle’s evolution by outlining the area of the four heart chambers at the pixel level for each frame. As mentioned, however, this requires a dataset for which manual segmentations are available for training. Understandably, since manual segmentation is not done routinely as part of the standard of care, it requires significant effort. We propose a method that sufficiently captures cardiac cycle information in longer TTEs, but recovers any useful information in shorter TTEs. We address the former requirement first and then the latter.

In [23] they demonstrate that by applying a NMF to TTEs, one can recover ED/ES phases as well as smoothed versions of video frames at ED and ES. Their work also suggests that all frames in an TTE video can be approximated by a linear combination of ED and ES frames. Consequently, the NMF yields a low-dimensional representation of the cardiac cycle, where the largest and smallest coefficients roughly correspond to ED and ES.

However, NMF-style decompositions work best for longer, high-quality TTEs. In practice, video lengths may be too short to estimate full ED/ES cycles. TTEs may also contain artifacts from various sources. For example, electromagnetic interference can produce artifacts that can be confused with atrial fibrillation, ventricular arrhythmias, and pacemaker dysfunction [39]. Other artifacts include reverberation artifacts when the ultrasound beam bounces multiple times between reflective surfaces before returning to the transducer [40]. (We refer the reader to [40] for more examples.) Nonetheless, we expect that the video should have *some* useful information about the cardiac cycle. Therefore, we choose to estimate and work with an encoding of the ultrasound that summarizes as much information as possible about the cardiac cycle.

Finally, fitting a NMF for each ultrasound would be computationally expensive, so we desire a faster and less expensive alternative. For standardized and non-negative data, a singular value decomposition (SVD) yields solutions that are similar to a NMF, but with the advantage that there are fast closed-form implementations for computing a SVD and importantly, that are compatible with gradient-based learning. With this in hand, we work with SVD rather than NMF. We present our approach as follows.

For simplicity, we describe a CNN taking one TTE clip as input. Let C denote the clip size, N the number of frames in the TTE, and P the height and width of a frame, where we take C=N. Let $X_{j}\in N^{C\times P\times P}$ be one TTE in our dataset. All our clips start at the first frame of the TTE. Note that our method does not depend on the choice of C and can accommodate arbitrary clip lengths. In contrast, [10] take multiple C=32 clips at random from TTEs. Moreover, they also keep every other frame in their clips, while we use every frame in our clip.

Let $Y_{j}\in R^{+}$ be the EF associated with $X_{j}$. We denote a dataset with D observations by ${\left\{X_{j},Y_{j}\right\}}_{j=1}^{D}$. Our model is parameterized by 2D spatial filters $W_{S}=\left\{w_{S}^{1},\ldots ,w_{S}^{L}\right\}$, 1D temporal filters $W_{T}=\left\{w_{T}^{1},\ldots ,w_{T}^{L}\right\}$, and bias parameters $B=\left\{b^{1},\ldots ,b^{L}\right\}$, where L is the number of layers. The full set of parameters is denoted by $\theta =W_{S}\cup W_{T}\cup B$.

Let $z^{0}$ be a pre-processed and normalized TTE (see Section 3.5 for pre-processing details) given as input to the model and let $z^{\ell }$ refer to the output of the $\ell^{\text{th}}$ layer. Define a sequence of activation functions as ${\left(a_{\ell }\right)}_{\ell =1}^{L}$. We work with ReLU activation functions $a_{\ell }(x):=\max(0,x),\;\forall x\in R$. Let ⋆ represent the (2+1)D convolution operation. For d∈N representing height and width, ⋆ computes a 1×d×d spatial 2D convolution followed by a t×1×1 temporal 1D convolution, where t∈N denotes the temporal dimension. We work with an R(2+1)D encoder over a 3 D CNN because it is less computationally expensive to train and performs at least as well as state-of-the-art 3D CNNs [21]. For simplicity, we refer to the $\ell^{\text{th}}$ filters of a (2+1)D convolution as $w^{\ell }$. The $\ell^{\text{th}}$ layer’s output is given by $z^{\ell }=f^{\ell }\left(z^{\ell -1}\right)=a_{\ell }\left(w^{\ell }⋆z^{\ell -1}+b^{\ell }\right),\;\forall \ell \in \{1,\ldots ,L-1\}$. Let p be a spatial average pooling layer.

Our network is comprised of an encoder E, a transformation ϕ that aggregates temporal features, and a decoder D. Taking $z^{0}$ as input, the network’s encoder is given by $E\left(z^{0}\right):=\left(p\left(f^{L-1}\right)\circ \cdots \circ f^{1}\right)\left(z^{0}\right)$. The next component of our network is a function ϕ for aggregating and weighting features. The decoder D takes ϕ‘s output as input to a linear layer. We write a generic version of our network as

(1) $$F_{\theta }\left(z^{0}\right)=\left(\underset{\text{Decoder}}{\underset{︸}{f^{L}}}\underset{\text{Feature Aggregation}}{\underset{︸}{\circ \;\phi \;\circ }}\underset{\text{Encoder}}{\underset{︸}{p\left(f^{L-1}\right)\circ \cdots \circ f^{1}}}\right)\left(z^{0}\right)\;\;=\left(D\circ \phi \circ E\right)(z^{0}).$$

#### Frame-Level Feature Aggregation.

Let M be the number of frame-level features. $\phi_{SVD}$ takes the encoder’s output as its input. Let $p\left(f^{L-1}\right):=o_{E}\in R^{M\times N}$ be the frame-level features produced by the encoder. $\phi_{SVD}$ estimates the coefficients associated with the first left-singular eigenvector of $o_{E}$, denoted by $h_{0}$. From there, we would like to use $\phi_{SVD}$ to weight $o_{E}$’s frame-level features by $h_{0}$. Finally, the decoder D uses the weighted frame-level features to estimate EF with a linear layer. We call the implementation of the model specified in (1) with $\phi_{SVD}$ *Spectral EchoNet*. Next, we motivate an extension of Spectral EchoNet to our final model, *SGS EchoNet*.

Consider an important detail that motivates our final choice of ϕ for our network. For two singular values associated with a single standardized TTE, $\sigma_{i},\sigma_{j}$ for i≠j, the following quantity is needed to compute backpropagated gradients:

(2) $$\frac{1}{\sigma_{i}-\sigma_{j}}.$$

For a small enough choice of ϵ>0, when $\left|\sigma_{i}-\sigma_{j}\right|\approx \epsilon$ we may have numerical instability in $\frac{1}{\sigma_{i}-\sigma_{j}}$. While we could choose some δ>0 large enough for $\frac{1}{\left(\sigma_{i}-\sigma_{j}\right)+\delta }$ to be numerically stable, it is not clear how to choose δ in a way that would guarantee numerical stability when the model is trained on one dataset versus another. Therefore, we looked for a transformation of $\phi_{\text{SVD}}$ that ensured smooth (and numerically stable) differentiation and chose the Scaled Gumbel Softmax (SGS) function, denoted $\phi_{SGS}.\;\phi_{SGS}$ composes a SGS transformation with the left singular eigenvector’s coefficients before following the rest of Spectral EchoNet’s operations.

Precisely, for a vector $V\in R^{m}$ with components $v_{i},\;i\in \{1,\ldots ,m\}$, the SGS function $s:R^{m}\mapsto R^{m}$ for the $i^{th}$ component is

(3) $$(s(V|\eta ,\tau ){)}_{i}=\eta \left[\frac{exp\left(\frac{v_{i}}{\tau }\right)}{\sum_{j=1}^{m}\;\;exp\left(\frac{v_{j}}{\tau }\right)}\right],\;(\forall i\in \{1,\ldots ,m\}).$$

A vector V transformed by s becomes vector $s_{0}$ with components $s_{i}:=(s(V|k,\tau ){)}_{i},\;i\in \{1,\ldots ,m\}.\;\tau$ and η are tuning parameters than can be learned from the data during training or tuned *ad hoc*.

Similar to $\phi_{SVD},\;\phi_{SGS}$ starts by taking $o_{E}$ as input and estimating its first left-singular eigenvector’s coefficients, $h_{0}$. From there, $\phi_{SGS}$ transforms $h_{0}$ by s, producing $s\left(h_{0}\right)=s_{0}$. In contrast to $\phi_{SVD},\;\phi_{SGS}$ uses $s_{0}$ to weight frame-level features. $\phi_{SGS}$ then passes the re-weighted frame-level features to the decoder D.

τ is the *temperature* parameter of the Gumbel-Softmax (GS) function. When η is fixed, as τ→0, the GS smoothly approaches the argmax function. Vectors sampled from the GS function for small τ approach one-hot encoded (or categorical) vectors. In our setting, this means as τ→0 the coefficient vector $s_{0}$ assigns higher weight to coefficients from V that were initially larger. Conversely, as τ→∞, the GS flattens inputs to have equal weight. Vectors sampled from the GS for large τ approach uniform vectors. Meanwhile, varying η scales the overall magnitude of all the GS values. Therefore, varying τ and η controls (at the dataset-level) how uniformly we weight inputs while increasing or decreasing the overall scale of the re-weighted inputs. For SGS EchoNet, τ and η let us re-weight SVD coefficients to have more pronounced peaks and troughs while controlling the overall scale of the coefficients. (See Figure 4 for examples.) Additionally, we note that for some videos the cardiac cycle may only be partially available due to video quality or video length. In these cases, SGS may also be tuned to amplify any weak signals before ϕ passes its output to the final linear layer.

We write SGS EchoNet as

(4) $$F_{\phi_{SGS}}\left(z^{0}\right)=\left(D\circ \phi_{SGS}\circ E\right)\left(z^{0}\right).$$

Note that our goal for any choice of ϕ is not to reproduce the cardiac cycle—the goal is to estimate coefficients that correspond to ED and ES for EF estimation as a way to avoid left-ventricular segmentation. See Figure 4 for examples of the coefficients used to identify ED and ES from a Stanford patient with low EF and one with high EF.

In summary, Spectral and SGS EchoNet both take full patient videos as input to an R (2+1)D encoder. We apply a SVD to the frame-level features in both networks. In Spectral EchoNet, we weight the frame-level features by its first left-singular eigenvector’s coefficients before passing them to the decoder. In SGS EchoNet, we apply the SGS transformation to the SVD coefficients before passing the weighted features to the decoder. For either ϕ, the result can be interpreted as the frame-level features re-weighted by coefficients corresponding to the ED/ES signal. In comparison, using a segmentation model to select clips near ED/ES is equivalent to assigning weights of 0 to frame-level features for ED/ES phases and assigning 1 to clips that are near them. Out of concern for numerical stability during training, however, we only move forward with training and testing SGS EchoNet.

## 5 Results

We open this section with results for the view classification model we used to identify AP4 TTEs. After discussing its performance, we discuss our signal recovery method. Finally, we present validation results and then an analysis of our model under two levels of view label noise on the external Duke Health dataset.

### 5.1 View Classification

**Fig. 3:**
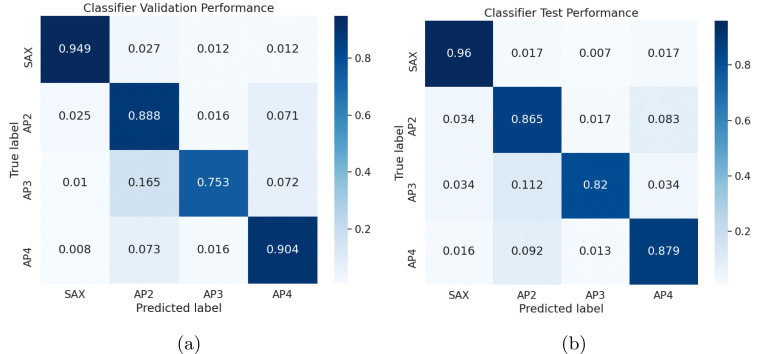
Confusion matrices for PROMISE Echo view classification normalized by row indicating true view classification rates. Validation accuracy (Left): 0.90. Test accuracy (Right): 0.89.

We trained a view classifier on the PROMISE Echo dataset and applied it to identify AP4 views in the CATHGEN dataset. Overall, the classifier showed strong validation performance and test performance. Overall accuracy is almost equivalent on test and validation sets. For test and validation sets, the classifier’s positive predictive value (PPV) is best for the short axis (SAX) and then AP4 views. Negative predictive value (NPV) is strong across all class predictions. The classifier’s recall and F1 scores are best for SAX followed by AP4 views. From Figure 3, we see that the class-specific classification accuracy for AP4 is approximately the same between test and validation, with ≈90% for AP4 classification accuracy on validation and ≈88% for AP4 classification accuracy on test.

Figure 3 also suggests that our classifier’s generalizes outside of its training and validation sets and can distinguish between AP4 and other views. When the true test label is AP4, we observe that roughly 12% of AP4 views were misclassified. While there is room for improvement, the focus of the our work is to classify labels in order to validate our model on a noisy external dataset. With this in mind, the AP4 row of Table 3 shows strong performance across PPV, NPV, Recall, and F1 score. This validates that our classifier is strong enough to identify views and reliably control view label noise.

### 5.2 ED/ES Phase Detection

**Fig. 4:**
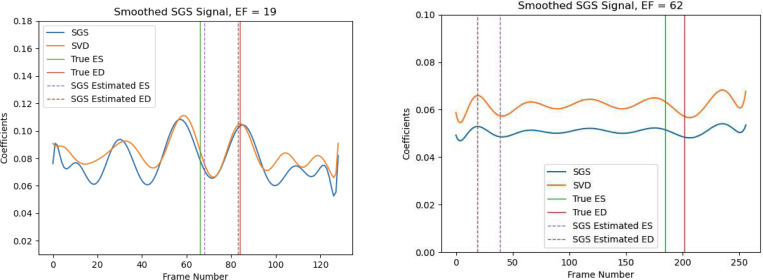
Example for ED/ES detection by SGS EchoNet. Peaks are identified using original, un-smoothed coefficients.

**Fig. 5:**
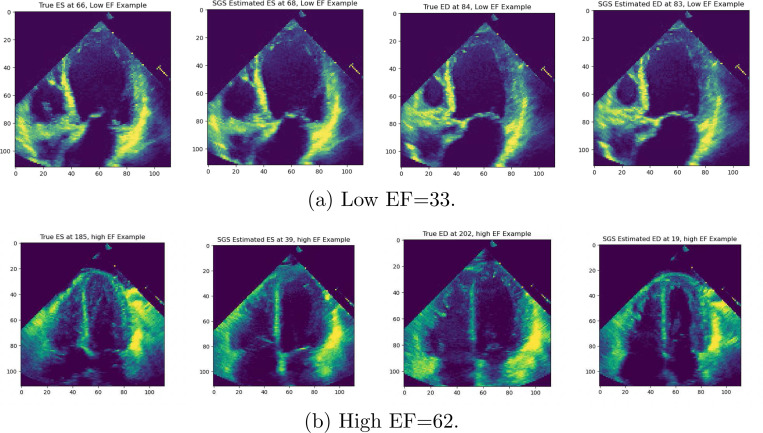
True *vs*. estimated ED and ES frames for Low *vs*. High EF.

Figure 4 shows two examples of patients’s estimated ED/ES phases from the Stanford dataset: one with low EF and one with high EF. Depending on the values of η and τ, we can see that the SGS transformation either diminishes or augments the cardiac signal estimated by the initial SVD. In the low EF example, the SGS transformation was set to augment the patient’s cardiac signal. $\phi_{SGS}$ and $\phi_{SVD}$ roughly coincide for the most pronounced peaks and troughs, but $\phi_{SVD}$’s curve flattens leading up to and away from ED and ES. $\phi_{SGS}$, however, amplifies the curve to bring out the underlying peaks and troughs outside of ED and ES. In the high EF example, the SGS transformation was set to diminish the patient’s cardiac signal. $\phi_{SGS}$ and $\phi_{SVD}$‘s curves follow the same path of peaks and troughs, but with $\phi_{SGS}$‘s curve shifted below $\phi_{SVD}$. Additionally, $\phi_{SGS}$ is nearly constant except for where estimated and true ED/ES occur. Finally, Figure 5 shows frames from the TTEs at true *vs*. estimated ED/ES.

### 5.3 Model Performances on Stanford and Duke Hospital Systems Datasets

In the following sections, we apply EchoNet No-Seg, EchoNet Dynamic, and SGS EchoNet to the Stanford Hospital System’s dataset and then the Duke Hospital System’s datasets. For each dataset, we follow the model-specific procedure for segmenting TTEs, if applicable, and estimating EF described in Section 4. We then apply the same models in the same way, to the extent possible, on the two Duke Hospital Systems datasets. Otherwise, we describe deviations when it is necessary to change anything about the model’s estimation procedure. For all models and all datasets, we compare model performances via MAE, RMSE, Pearson’s r, mean relative absolute error (MRAE), AUC, and the proportion of estimates that are over or under a pre-specified threshold. We set the threshold for over- or under-prediction according to studies in inter-observer variation in human assessment of EF. Empirically, inter-observer variation for EF assessment ranges from 7.6% to 13.9% [4, 11]. After rounding to 8% and 14%, we set the midpoint between this range, 11%, as the threshold. This means that for a true EF value of 50, an estimate greater than 55.5 would be classified as an over-prediction, and an estimate of 44.5 would be classified as an under-prediction. In Tables 4−6, $p_{\text{over}}$ and $p_{\text{under}}$ denote the average number of estimates that were classified as over- vs. under-predicted in the dataset, respectively. In all figures, we graphically represent $p_{\text{over}}$ by points colored coral and $p_{\text{under}}$ by points colored lime green. In all tables, we report confidence intervals in parentheses. Confidence intervals were computed using 10,000 bootstrapped samples and by obtaining 95^th^ percentile ranges for each prediction.

In Tables 5 and 6, we denote EchoNet Dynamic with Beat-by-Beat estimates as EchoNet Dynamic: ByB and EchoNet Dynamic with averaging across all clip-level estimates as EchoNet Dynamic: Avg.

### 5.4 Validation on Stanford Hospital System’s Dataset

**Fig. B1:**
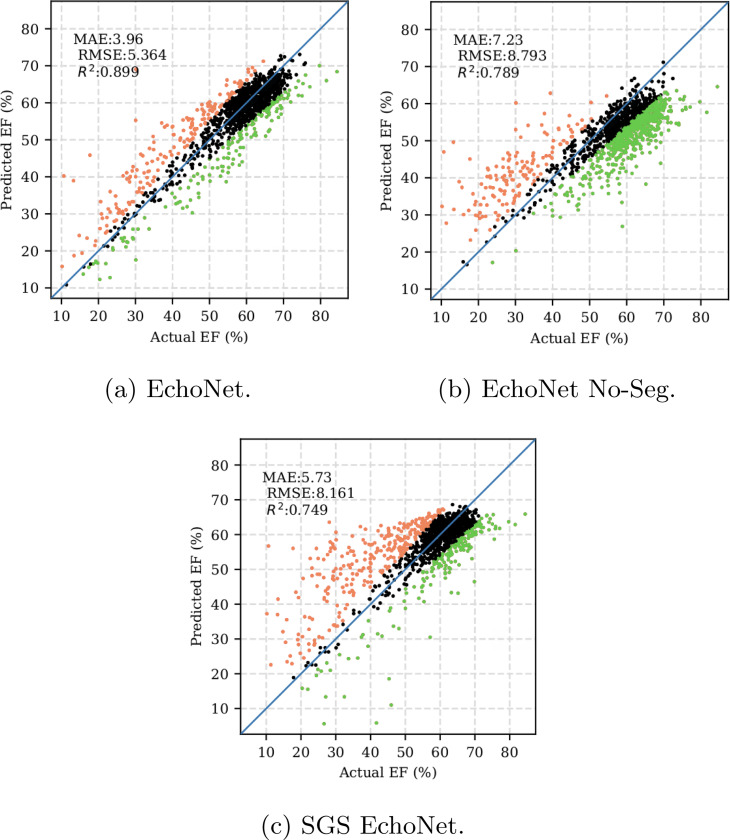
Comparisons on Stanford data.

Table 4 shows that on the Stanford dataset, EchoNet performs best overall in mean absolute error (MAE), root-mean-square error (RMSE), correlation, and area under the ROC curve (AUC), followed by the SGS model and EchoNet No-Seg. In the absence of any ED/ES identification model, EchoNet No-Seg’s performs worst overall. Comparing EchoNet No-Seg’s RMSE to the other models, we can also see that the ED/ES identification plays a significant role in reducing variability of estiamtes. All models show a strong correlation between true vs. predicted EF. Looking only at Table 4, EchoNet No-Seg appears to offer moderate performance, but Figure B1 shows that EchoNet No-Seg’s ED values rotate roughly 15° off the 45° line as it drifts to the mean of EF=55.6 in the absence of a segmentation model. While EchoNet No-Seg does not perform poorly, this confirms that ED/ES identification plays a key role in calibrating the EF model’s estimates, and that our proposed ED/ES identification approach enhances performance in the absence of a segmentation model.

### 5.5 Validation on Duke Health Care Dataset

Now we compare our models using the noisy real-world dataset from Duke Healthcare Systems as our validation set. Note that there were no segmentation labels for TTEs available for the Duke data, so we were unable to refine EchoNet’s segmentation model on Duke TTEs. Therefore, we ran EchoNet’s segmentation model as-is and used the resulting segmentations in EchoNet’s workflow for generating EF predictions.

#### Model Comparisons under Varying Noise Levels

We compare model performance when the classification threshold for an *AP4* video is set at *p*_Ap4_≥0.7 and *p*_Ap4_≥0.99. We work with our SGS model due to its numerical stability during training and greater flexibility (see Section 4). Each model has *only* been trained and validated on the Stanford data before being applied to the Duke test set, so there is no refinement for any model. The one constraint for these data is that the set of usable TTEs is small, even though the set of initially available Duke TTEs starts out large. Despite its small size, however, this allows us to study our models on a noisier external dataset.

In Tables 5 and 6, we denote EchoNet Dynamic with Beat-by-Beat estimates as EchoNet Dynamic: ByB and EchoNet Dynamic with averaging across all clip-level estimates as EchoNet Dynamic: Avg. While EchoNet Dynamic: ByB *looks* as though it initially performs well, we highlight EchoNet Dynamic: ByB metrics with the superscript ‡ because they are are inflated in the following sense.

In order to estimate EF from beat-to-beat within a TTE, EchoNet Dynamic relies on its segmentation model and peak-finding algorithm to identify all clips that correspond to ED or ES. After identifying ED and ES phases, EchoNet Dynamic *drops all other clip-level estimates*. Finally, EchoNet Dynamic averages the remaining clip-level EF estimates to produce a final EF estimate for the TTE. However*, if EchoNet Dynamic does not detect any ED or ES phases, it drops every clip in the TTE and fails to produce an EF estimate*. Consequently, the metrics highlighted by ‡ in Tables 5 and 6 only summarize the TTEs for which EchoNet Dynamic: ByB could produce estimates. This is misleading because EchoNet Dynamic *failed* to produce EF estimates for the vast majority of TTEs in the Duke datasets. Specifically, EchoNet Dynamic: ByB was *only* able to produce estimates for 5 out of 26 TTEs in the CATHGEN data with *p*_APC_ ≥.99, and 26 out of 155 in in the CATHGEN data with *p*_APC_≥.70. Due to the small number of estimates, we do not compute bootstrap confidence intervals for EchoNet Dynamic: ByB in Table 5.

Therefore, *while the results for EchoNet Dynamic: ByB initially look strong in Tables 5 and 6, EchoNet Dynamic: ByB actually struggled to produce EF estimates*. In contrast, EchoNet Dynamic: Avg does not drop any clips, so it was able to produce estimates for each TTE. However, it performed worse across the board than EchoNet No-Seg, so we focus the rest of our discussion on comparing EchoNet No-Seg and SGS EchoNet.

Examining SGS EchoNet’s performance, Tables 5 and 6 show that SGS EchoNet outperforms EchoNet No-Seg in nearly all metrics. Comparing SGS EchoNet and EchoNet No-Seg’s estimates in Figure B3, we see that SGS EchoNet’s estimates are strongly anchored about the 45° guide line. While we observe a strong linear relationship between EchoNet No-Seg’s estimates and true EF values, its estimates are miscalibrated and drift back toward the mean EF value, as we observed in the Stanford data in Figure B1.

When $p_{A4C}=0.7$, Table 6 shows MAE and RMSE are lower for SGS EchoNet than EchoNet No-Seg and EchoNet Dynamic, but within error of each other. At first glance, SGS EchoNet also shows worse correlation and AUC than EchoNet No-Seg, but stronger mean relative absolute error (MRAE) and a better balance of over- and under-predictions ($p_{\text{over}}$ and $p_{\text{under}}$, respectively).

**Fig. B2:**
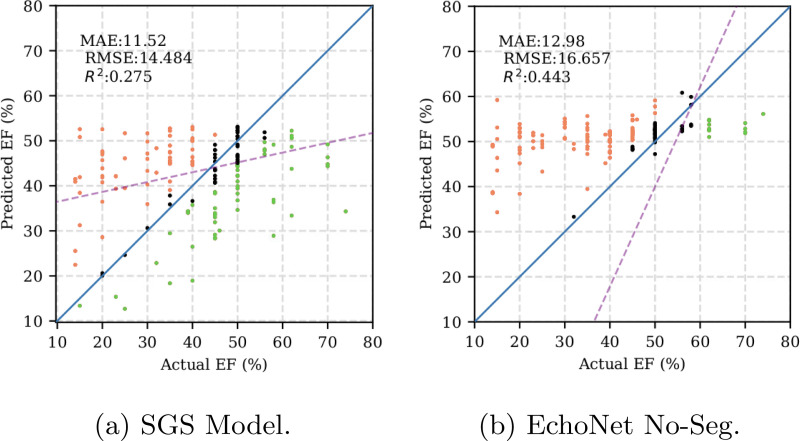
SGS EchoNet *vs*. EchoNet No-Seg with $p_{AP4}\geq 0.7$.

**Fig. B3:**
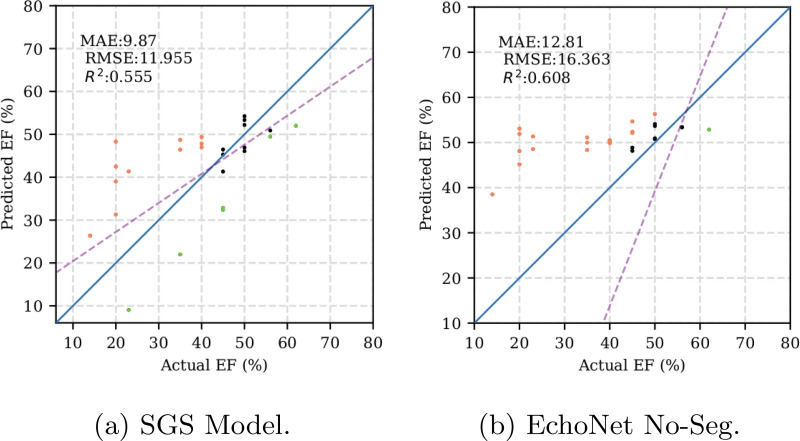
SGS EchoNet *vs*. EchoNet No-Seg with $p_{\text{AP4}}\geq 0.99$.

Visually inspecting Figure B2, however, we see EchoNet No-Seg is merely predicting the mean of the dataset, which mirrors the miscalibration we see in Figure B3. In contrast, SGS EchoNet’s estimates in Figure B3 show less drifting toward the mean and better anchoring about the 45° line. Looking at Figures B3 and B2 together, we note that EchoNet No-Seg becomes miscalibrated regardless of the level of $p_{AP4}$, whereas the SGS model remains more centered on the 45° line throughout. Altogether, this may suggest that the metrics in Figure B2 are rewarding EchoNet No-Seg’s estimates simply because they are clustered closer together, even though they are less useful than SGS EchoNet’s estimates.

To demonstrate that the metrics for EchoNet No-Seg are rewarding clustering despite miscalibration, we estimated the offset and slope associated with each model by Siegel’s robust median regression procedure [41, 42]. We plot the estimated regression line as a dashed, purple line in Figures B3 and B2. For all models, an offset closer to 0 and a slope closer to 1 is better.

In Table 7 we see that the offset and slope estimates capture the disorientation in EchoNet No-Seg’s estimates vs. SGS EchoNet’s. For all datasets, SGS EchoNet is anchored better on the 45° line, and as a result, SGS EchoNet’s estimated offset is closer to 0 and its estimated slope is closer to 1 than EchoNet No-Seg for every dataset.

## 6 Conclusion

In this work, we presented a more parsimonious and robust video-based DL model that achieves comparable performance to the state-of-the-art approach. SGS EchoNet achieves this by applying an SGS-transformed SVD after its pooling layer to recover ED/ES phases. In doing so, SGS EchoNet demonstrates that it is possible to estimate EF well without a segmentation model. Importantly, we also showed that SGS EchoNet is robust to moderate noise on an external hospital system’s dataset. Owing to the small size of usable TTEs on the external validation set, rigorously studying our models and other candidates on larger external validation sets remains a direction for future work. Finally, we saw that SGS transformation can be tuned *ad hoc* in the absence of a new training set. In this way, when it is too expensive to acquire hand-traced TTEs or too difficult to train a high-quality segmentation model, it may be possible to use the SGS transformation as an alternative to a segmentation model.

A limitation of this work is the small sample size of TTEs that were available for the external validation set. While we were able to provide some insight into our model and EchoNet Dynamic’s behavior at various levels of noise, our analysis should be taken as the first step toward studying our model and other candidates on larger external validation sets. Another substantial limitation was the numerical instability introduced by the SVD transformation without the SGS transformation. While the SGS transformation assuages this problem, the literature on SVD-related applications is vast, so it is possible that other forms of SVD (or other estimation procedures) for SVD could resolve this issue. For future work, two straightforward directions are a deeper study into exactly when SVD may be numerically unstable for decomposing TTE features and an examination of how models handle input noise on larger TTE datasets. A fruitful direction would also be to study other classes of smooth transformations of SVD apart from SGS. We speculate that it may be possible to work with different transformations of the SVD features to estimate other clinically important quantities that are a function of end diastole and end systole phases of the heart. This would depend on clinicians articulating other meaningful quantities, so it will be important to work closely with cardiovascular clinicians to determine what classes of transformation may be promising.

## Figures and Tables

**Table 1:** Total number of videos by category for external validation set.

CATHGEN AP4 TTEs	# Videos
Total	1,044
*p*_AP4_ ≥ 0.7	540
*p*_AP4_ ≥ 0.8	423
*p*_AP4_ ≥ 0.9	286
*p*_AP4_ ≥ 0.99	80
EF= 55	630
With Doppler	518
EF≠ 55, *p*_AP4_ ≥ 0.7, no doppler	128
EF≠ 55, *p*_AP4_ ≥ 0.99, no doppler	28

**Table 2:** Breakdown of TTE views used to train view classifier.

PROMISE TTE Views	# Views	Proportion
AP4	1,901	0.334
AP3	309	0.054
AP2	1,868	0.328
PSAX	1,622	0.285

**Table 3:** Classifier metrics on validation and test sets.

	Validation					Test				

View	PPV	NPV	Recall	F1	Support	PPV	NPV	Recall	F1	Support
SAX	.96	.98	.95	.96	334	.93	.99	.96	.95	300
AP2	.84	.96	.89	.87	322	.86	.94	.86	.86	348
AP3	.83	.98	.75	.79	97	.85	.98	.82	.83	89
AP4	.91	.95	.90	.91	384	.90	.94	.88	.89	371

**Table 4:** Performance on Stanford data.

Model	MAE	RMSE	*r*	MRAE	AUC	$p_{\text{over}}$	$p_{\text{under}}$
EchoNet No-Seg	7.23 (7.08, 7.39)	8.79 (8.64, 9.96)	.79 (.78, .80)	.268 (.263, .274)	.925	.111	.451
EchoNet Dynamic	3.92 (3.76, 4.08)	5.36 (5.00, 5.52)	.90 (.89, .91)	.284 (.278,.290)	.963	.138	.120
SGS EchoNet	5.73 (5.47, 6.00)	8.16 (7.74, 8.61)	.75 (.72, .78)	.137 (.126,.149)	.892	.224	.14

**Table 5:** CATHGEN Data: $p_{A4C}\geq 0.99$.

Model	MAE	RMSE	*r*	MRAE	AUC	$p_{\text{over}}$	$p_{\text{under}}$
EchoNet No-Seg	12.81 (9.63, 16.22)	16.36 (12.82, 19.51)	.61 (.34, .79)	.511 (.328, .708)	.812	.650	0.04
EchoNet Dynamic: ByB^‡^	7.86	9.91	.85	.177	1.0	.571	0.0
EchoNet Dynamic: Avg	13.51 (10.21, 17.02)	17.11 (13.08, 20.97)	.66 (.41, .84)	.453 (.297, .635)	.899	.808	0.0
SGS EchoNet	9.87 (7.76, 12.07)	11.96 (9.44, 14.36)	.55 (.35, .73)	.362 (.247, .486)	.870	.423	0.231

‡: ($\frac{21}{26}$ dropped).

**Table 6:** CATHGEN Data: $p_{A4C}\geq 0.70$

Model	MAE	RMSE	*r*	MRAE	AUC	$p_{\text{over}}$	$p_{\text{under}}$
EchoNet No-Seg	12.98 (11.61, 14.38)	16.66 (15.14, 18.10)	.44 (.33, .55)	.510 (.424, .591)	.825	.639	0.077
EchoNet Dynamic: ByB^‡^	12.02 (9.27, 15.02)	15.04 (11.30, 18.48)	.70 (.51, .86)	.387 (.243, .567)	.96	.731	0.0
EchoNet Dynamic: Avg	14.62 (13.23, 16.07)	18.11 (16.46, 19.68)	.57 (.48, .65)	.510 (.434, .588)	.862	.768	0.039
SGS EchoNet	11.52 (10.40, 12.68)	14.48 (13.15, 15.74)	.28 (.15, .39)	.390 (.331, .454)	.640	.387	0.361

‡: ($\frac{129}{155}$ dropped).

**Table 7:** Siegel’s offset and slope estimation.

	Stanford		CATHGEN $p_{A4C}\geq 0.99$		CATHGEN $p_{A4C}\geq 0.70$	
Model	Offset	Slope	Offset	Slope	Offset	Slope
EchoNet No-Seg	3.522	1.032	−87.942	2.543	−70.697	2.213
SGS EchoNet	1.116	.978	12.93	.676	30.266	.295

## Data Availability

One dataset we use is publicly available EchoNet Dynamic dataset, made available through this website: https://aimi.stanford.edu/datasets/echonet-dynamic-cardiac-ultrasound. The other dataset is a Duke Health Care dataset called the CATHeterization GENetics (CATHGEN) dataset, which is not publicly available due to containing sensitive health data. In addition to genetic information, the CATHGEN biorepository collected clinical data from patients that included medical history, lifestyle factors, and follow-up information on cardiovascular events. For full details, we refer readers to “A Guide for a Cardiovascular Genomics Biorepository: the CATHGEN Experience” in the Journal of Cardiovascular Translational Research.

## Footnotes

1 Technically, CNNs perform semantic segmentation, i.e., pixel-wise classification of input images, but we will refer to the task generically as segmentation.

## Appendix A Model Training

For all models, the weights from the epoch with the lowest validation loss was selected for final testing.

### A.1 EchoNet Dynamic

We train EchoNet Dynamic based on details provided by [10]. We initialize EchoNet’s R(2+1)D model with pretrained weights from the Kinetics-400 dataset [36]. Next, we train EchoNet’s EF model using stochastic gradient descent with initial learning rate 0.0001, momentum 0.9, batch size of 16 on 45 epochs, and a squared error loss function. The biases of the network were initialized to the mean EF of the dataset, 55.6. The learning rate was decreased by a factor of 0.1 every 15 epochs for a total of 45 epochs. The model took 32 -frame video clips as input with a sampling period of 2 to sample every other frame in the ultrasound. Finally, during training on the Stanford Hospitals data, following [10], we augment dataset size and increase training clip variation by padding training video clips with 12 pixels on each side.

Following [10], we initialize EchoNet’s DeepLabV3 segmentation model with random weights and optimize using stochastic gradient descent with a pixel-level binary cross-entropy loss function. We used a learning rate of 0.00001, momentum of 0.9, and batch size of 20 for 50 epochs.

### A.2 Spectral & SGS EchoNet

We train SGS EchoNet under a Huber loss function with $\phi_{SGS}$ active during training. Additionally, we provide SGS EchoNet with 64 -frame video clips as input in contrast to EchoNet’s subsampled 32-frame clips. We train with initial learning rate 0.0001 over 30 epochs with the learning rate decreased by a factor of 0.1 every 10. Unlike EchoNet Dynamic, the biases of our network were *not* initialized to the mean EF of the dataset, and our Huber loss function computed loss with respect to the true EF minus the mean EF of the Stanford TTEs, *i.e*., 55.6.
